# BECLIN1 Is Essential for Podocyte Secretory Pathways Mediating VEGF Secretion and Podocyte-Endothelial Crosstalk

**DOI:** 10.3390/ijms23073825

**Published:** 2022-03-30

**Authors:** Tillmann Bork, Wei Liang, Oliver Kretz, Simon Lagies, Kosuke Yamahara, Camila Hernando-Erhard, Martin Helmstädter, Christoph Schell, Bernd Kammerer, Tobias B. Huber

**Affiliations:** 1 Department of Medicine IV, Faculty of Medicine, University of Freiburg, 79106 Freiburg, Germany; tillmann.bork@uniklinik-freiburg.de (T.B.); camila_hernando@yahoo.de (C.H.-E.); martin.helmstaedter@uniklinik-freiburg.de (M.H.); 2 Division of Nephrology, Renmin Hospital of Wuhan University, Wuhan 430060, China; liangweiwhu@foxmail.com; 3 III Department of Medicine, University Medical Center Hamburg-Eppendorf, 20246 Hamburg, Germany; o.kretz@uke.de; 4 Centre for Integrative Signalling Analysis, University of Freiburg, 79104 Freiburg, Germany; simon.lagies@zbsa.uni-freiburg.de (S.L.); bernd.kammerer@zbsa.uni-freiburg.de (B.K.); 5 Institute of Organic Chemistry, University of Freiburg, 79104 Freiburg, Germany; 6 Department of Medicine, Shiga University of Medical Science, Otsu 520-2192, Japan; yamaharakosuke@gmail.com; 7 Institute of Surgical Pathology, Faculty of Medicine, University of Freiburg, 79106 Freiburg, Germany; christoph.schell@uniklinik-freiburg.de; 8 BIOSS Centre for Biological Signalling Studies, Albert-Ludwigs-University Freiburg, 79104 Freiburg, Germany

**Keywords:** podocyte, BECLIN1, autophagy, secretory pathway, Golgi network, VEGF, glomerulosclerosis

## Abstract

Vascular endothelial growth factor A (VEGFA) secretion from podocytes is crucial for maintaining endothelial integrity within the glomerular filtration barrier. However, until now, the molecular mechanisms underlying podocyte secretory function remained unclear. Through podocyte-specific deletion of BECLIN1 (*ATG6* or *Becn1*), a key protein in autophagy initiation, we identified a major role for this molecule in anterograde Golgi trafficking. The *Becn1*-deficient podocytes displayed aberrant vesicle formation in the *trans-*Golgi network (TGN), leading to dramatic vesicle accumulation and complex disrupted patterns of intracellular vesicle trafficking and membrane dynamics. Phenotypically, podocyte-specific deletion of *Becn1* resulted in early-onset glomerulosclerosis, which rapidly progressed and dramatically reduced mouse life span. Further, in vivo and in vitro studies clearly showed that VEGFA secretion, and thereby endothelial integrity, greatly depended on BECLIN1 availability and function. Being the first to demonstrate the importance of a secretory pathway for podocyte integrity and function, we identified BECLIN1 as a key component in this complex cellular process. Functionally, by promoting VEGFA secretion, a specific secretory pathway emerged as an essential component for the podocyte-endothelial crosstalk that maintains the glomerular filtration barrier.

## 1. Introduction

Body fluid homeostasis largely depends on an intact glomerular filtration barrier, which is a unique structure essential for water excretion and macromolecule retention. This barrier is formed with three layers: the fenestrated endothelium, the glomerular basement membrane and highly specialized postmitotic epithelial cells called podocytes [1]. Podocytes assemble in a complex network with their interdigitating foot processes closely surrounding glomerular capillaries. In addition to their structural contribution to the glomerular filtration barrier (through their interdigitating foot processes and by spanning the slit diaphragm), podocytes show paracrine function, as indicated by a recent description of podocytes as the main glomerular sources of vascular endothelial growth factor A (VEGFA), which promotes complex podocyte-endothelial cell crosstalk [2]. In the kidney, VEGF has been shown to be indispensable for glomerular development because it regulates the proliferation and survival of recruited endothelial cells, and, in mature glomeruli, VEGF preserves endothelial function by maintaining endothelial fenestration [2]. Increased levels of VEGF, a candidate secretory protein, clearly suggest podocyte secretory activity, but, until now, the molecular mechanisms underlying podocyte secretory function remained unclear.

Two major vesicle trafficking pathways have been proven to be critical for podocyte function: endocytosis and autophagy [3,4]. Autophagy (in Greek, self-eating) is an intracellular process through which misfolded proteins and dysfunctional organelles are enclosed in double-membrane vesicles that fuse with lysosomes, forming compartments where vesicle contents are degraded to provide recycled amino acids and membrane components for cellular self-renewal [5,6]. Podocytes exhibit extraordinarily high autophagy and autophagic flux levels, which are regulated by a cell-specific signalling hub [7]. Key autophagy-related (ATG) proteins are enriched in podocytes; however, a reanalysis of the molecular structure and interactome of certain ATG proteins has strongly suggested additional, or even primary, involvement of ATGs in other cellular processes. One ATG candidate with multiple function potential is the BECLIN 1 protein.

BECLIN1 has been identified as a BCL-2-interacting protein [8] and the mammalian orthologue of yeast ATG6, a key autophagy molecule that forms complexes with the PI3 kinase VPS34 [9], thereby promoting autophagy initiation [10]. Furthermore, numerous studies have revealed a complex and dynamic network of BECLIN1 interaction partners (such as UV irradiation resistance-associated gene (UVRAG) and AMBRA1) that regulate the BECLIN 1–VPS34 complex, thereby modulating autophagy; however, other non-autophagy-related functions also seem to be outcomes of these interactions, particularly because the interaction partners of VPS34 and VPS15 exert a profound impact on vesicle trafficking and endocytosis [11].

Through our study, we aimed to characterize the molecular functions of BECLIN1 in podocytes and to identify new functions of this molecule in addition to its role in autophagy. Through podocyte-specific deletion of *Becn1*, we developed, for the first time, an in vivo model for successfully targeting the secretory pathway of podocytes. The results suggested that *Becn1* contributes considerably to glomerular integrity and function by mediating VEGF secretion.

## 2. Results

### 2.1. Beclin1 Is Essential for Podocyte Integrity and Glomerular Podocyte Network Architecture

**Figure 1:**
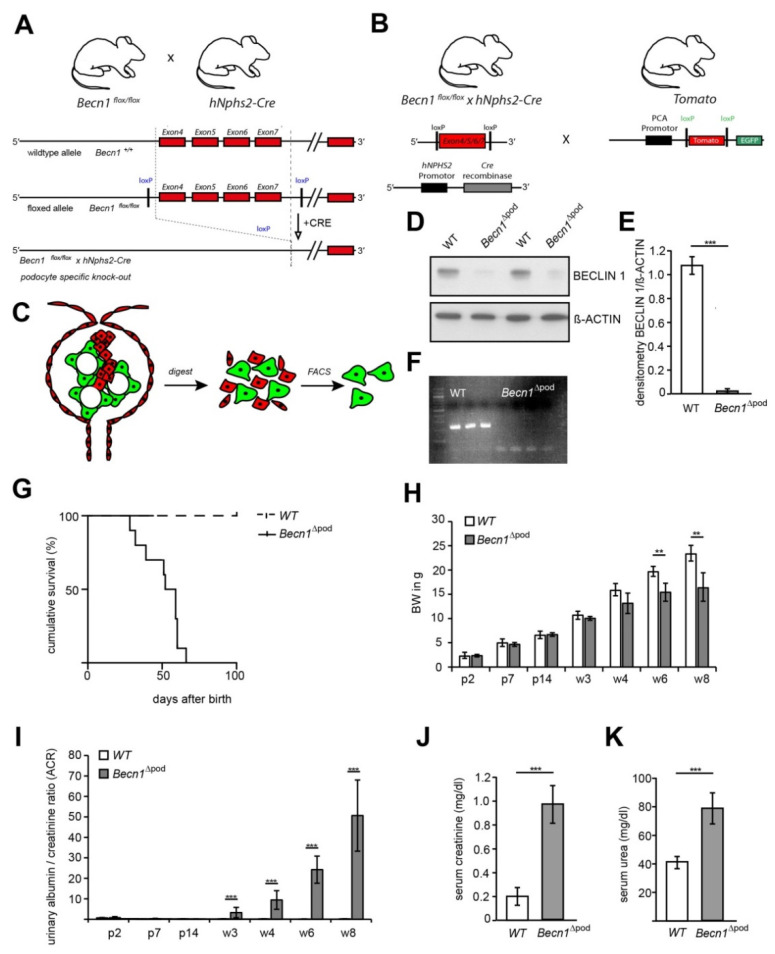
Podocyte-specific knockout of *Becn1* expression. (**A**) Schematic showing the generation of *Becn1*^Δpod^ mice by crossing *Becn1^flox/flox^* mice with *hNphs2-Cre* mice; (**B**) Schematic showing the generation of fluorescence-labelled podocytes by crossing *Becn1^flox/flox^ × hNphs2-Cre* mice with *Tomato/EGFP* reporter mice; (**C**) Schematic showing the ex vivo isolation of podocytes after glomeruli isolation, digestion and mechanical shearing and subsequent fluorescence-activated cell sorting (FACS); (**D**) Western blotting was performed to determine the abundance of BECLIN1 in ex vivo isolated primary podocytes; (**E**) Densitometry data obtained from samples shown in (**D**) (*** ≤ 0.001); (**F**) PCR was performed to identify *Becn1* deletion using a DNA template obtained from isolated *Becn1*^Δpod^ and *WT* podocytes. (**G**) Survival analysis of *Becn1*^Δpod^ and *WT* mice (n = 30 each genotype); (**H**) Body weight of *Becn1*^Δpod^ and *WT* mice through week 8 (** ≤ 0.01); (**I**) Urinary albumin/creatinine ratio in *Becn1*^Δpod^ and *WT* mice through week 8 (** ≤ 0.01 and *** ≤ 0.001, respectively); (**J**) Serum creatinine (mg/dL) in *WT* and *Becn1*^Δpod^ mice *** ≤ 0.001); (**K**) Serum urea (mg/dL) in *WT* and *Becn1*^Δpod^ mice (*** ≤ 0.001).

BECLIN1 expression is upregulated in chronic kidney disease (CKD) (Figure S1A,B), and follows the expression pattern of numerous other genes contributing to autophagy (Figure S1C) [12], suggesting the involvement of BECLIN1 in this important cellular degradation pathway. However, the molecular structure of BECLIN1 and its complex interactome has raised the possibility that BECLIN1 promotes cellular mechanisms, in addition to autophagy, in healthy podocytes. To elucidate the function of BECLIN1, mice harbouring *Becn1* floxed alleles (*Becn1^flox/flox^*) were crossed with mice expressing *Cre* recombinase under the control of the *hNphs2* promoter. The resulting mice harboured a podocyte-specific mutation in which the *Becn1* gene was deleted (*Becn1*^∆pod^) (Figure 1A). For ex vivo podocyte isolation, *Becn1^flox/flox^*;*hNphs2-Cre* mice were additionally crossed to mice harbouring a reporter *Tomato/EGFP* allele, which resulted in green fluorescence emitting from podocytes (Figure 1B). To confirm *Becn1* deletion ex vivo, podocyte isolation was performed in two-week-old mice after glomeruli isolation using magnetic beads followed by digestion and mechanical shearing. After fluorescence-activated cell sorting (FACS), a pure podocyte fraction was obtained (Figure 1C). Loss of the BECLIN1 protein was confirmed by Western blot analysis (Figure 1D, with quantification shown in Figure 1E), and successful recombination resulting in *Becn1* gene deletion was demonstrated by PCR (Figure 1F). Phenotypically, the mice were born at a normal Mendelian ratio and without any obvious abnormalities at birth. Subsequently, the mice with the podocyte-specific *Becn1* deletion showed a dramatically reduced life span (Figure 1G) and increased growth retardation, as indicated by a significantly reduced body weight by postnatal week 6 (Figure 1H). *Becn1*^∆pod^ mice developed rapidly progressive proteinuria starting at postnatal week 3 (Figure 1I), followed by impaired renal function after eight weeks, as indicated by an increased serum creatinine level (Figure 1J) and serum urea level (Figure 1K).

**Figure 2:**
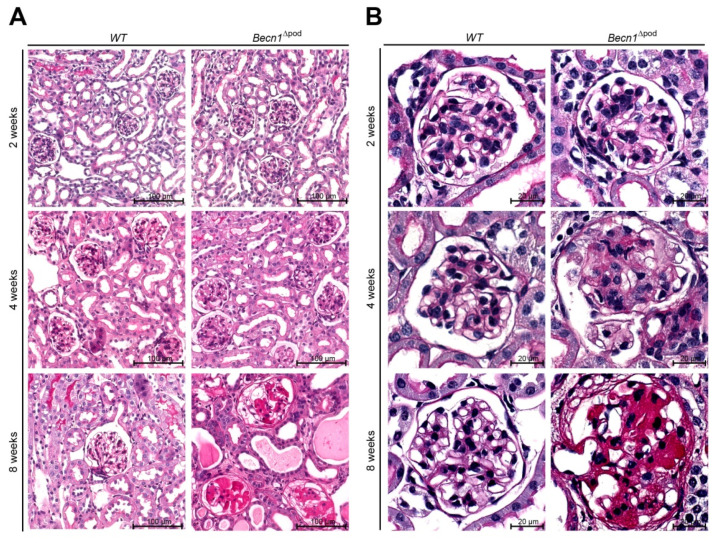

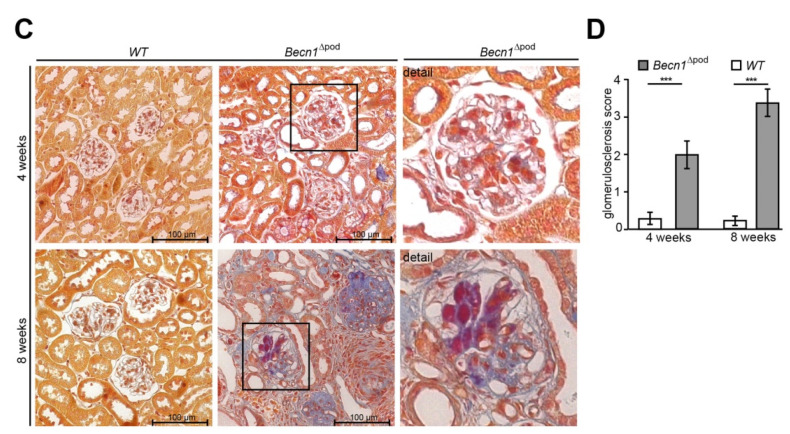
Podocyte-specific knockout of *Becn1* leads to glomerular scarring. (**A**) Kidney sections obtained from 2-week-old, 4-week-old and 8-week-old WT and *Becn1*^Δpod^ mice stained with haematoxylin-eosin solution; (**B**) Glomeruli from kidney sections obtained from 2-week-old, 4-week-old and 8-week-old WT and *Becn1*^Δpod^ mice stained with haematoxylin-eosin solution; (**C**) Kidney sections and magnified glomeruli obtained from 4-week-old and 8-week-old WT and *Becn1*^Δpod^ mice stained with acid-fuchsin, orange-G, and aniline blue solution; (**D**) Glomerulosclerosis score calculated using the method of el Nahas in 4-week-old and 8-week-old WT and *Becn1*^Δpod^ mice (*** ≤ 0.001).

With respect to proteinuria, kidney sections showed no differences between two-week-old mice; however, in four-week-old mice, glomerulosclerosis became apparent in the *Becn1*^∆pod^ mice, furthering the rapid progression to excessive glomerular scarring, tubular cast formation and tubular dilatation by postnatal week 8 (Figure 2A,B, quantification is shown in Figure 2D as described by el Nahas et al. [13]). The development of glomerular lesions due to podocyte-specific *Becn1* deficiency was accompanied by glomerular and interstitial fibrosis (Figure 2C). Scanning electron microscopy (SEM) revealed disrupted foot process formation in *Becn1*-deficient podocytes with rarefication of secondary processes and a disorganized foot process network (Figure S2A), results that were consistent with our functional experiments showing increased proteinuria.

### 2.2. Autophagosomes Form Independent of BECLIN1 Expression

**Figure 3:**
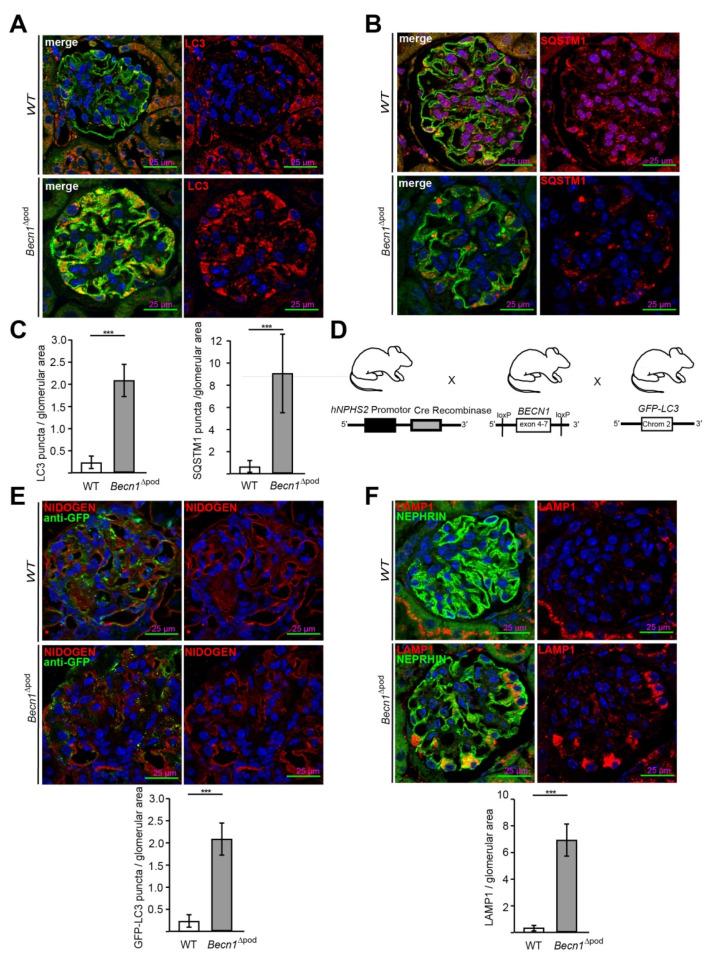
*Becn1*^Δpod^ mice displaying a high number of autophagosomes with an increased lysosomal compartment. (**A**) Representative image showing immunofluorescence staining for NEPHRIN (green) and LC3 (red) in kidney sections obtained from 4-week-old WT and *Becn1*^∆pod^ mice; (**B**) Representative image showing immunofluorescence staining for NEPHRIN (green) and SQSTM1 (red) in kidney sections obtained from 4-week-old WT and *Becn1*^∆pod^ mice; (**C**) Quantification of LC3 and SQSTM1 aggregates in kidney sections obtained from 4-week-old WT and *Becn1*^∆pod^ mice (n = 6). (**D**) Schematic showing the generation of mice with podocyte-specific deletion of *Becn1* and transgenic mice carrying fluorescence-labelled LC3 (*GFP-LC3*); (**E**) Representative immunofluorescence staining for NIDOGEN (red) and anti-GFP (green) in kidney sections obtained from 4-week-old *GFP-LC3* and *GFP-LC3* × *Becn1*^∆pod^ mice. Quantification of GFP-LC3 puncta (n = 6, *** ≤ 0.001); (**F**) Representative image showing immunofluorescence staining for NEPHRIN (green) and LAMP1 (red) in kidney sections obtained from 4-week-old WT and *Becn1*^∆pod^ mice. Quantification of the LAMP1 signal (n = 8, *** ≤ 0.001).

As part of the autophagy initiation complex, BECLIN1 plays a crucial role in autophagy. In this study, the levels of LC3 **(**Figure 3A) and SQSTM1 (Figure 3B) in sections obtained from four-week-old wild-type (WT) and *Becn1*^∆pod^ mice were assessed by immunofluorescence. A lack of BECLIN1 led to the massive accumulation of LC3 which formed aggregates in podocytes. Furthermore, a Western blot analysis of glomerular lysates obtained by perfusion with magnetic beads confirmed an increased level of lipidated LC3 (named LC3-II), indicating that LC3 was integrated into the autophagosomal membrane at high levels (Figure S2B). In parallel, assessment of the autophagy adaptor protein SQSTM1 revealed large SQSTM1 aggregates in *Becn1*-deficient podocytes. The accumulation of both LC3 and SQSTM1 (Figure 3C) clearly indicated altered autophagy in the *Becn1*^∆pod^ mice. Because BECLIN1 has a known function in autophagy initiation, the *Becn1*^∆pod^ mice were crossed with mice harbouring a transgene for *GFP-LC3* to establish mice in which autophagosome formation could be assessed in vivo (Figure 3D). Immunofluorescence analysis of tissue sections obtained from four-week-old mice showed an increased number of formed GFP-LC3 puncta (Figure 3E) in the *Becn1*^∆pod^ mice, and thereby, autophagosome formation was ongoing despite the presumed disruption in the autophagy initiation complex due to the lack of BECLIN1. The increase in LC3 and SQSTM1 aggregates was accompanied by an increase in the number and size of lysosomal compartments, as indicated by a high level of LAMP1-positive fluorescence in podocytes lacking *Becn1* (Figure 3F).

### 2.3. Loss of BECLIN1 Leads to Vesicle Accumulation

**Figure 4:**
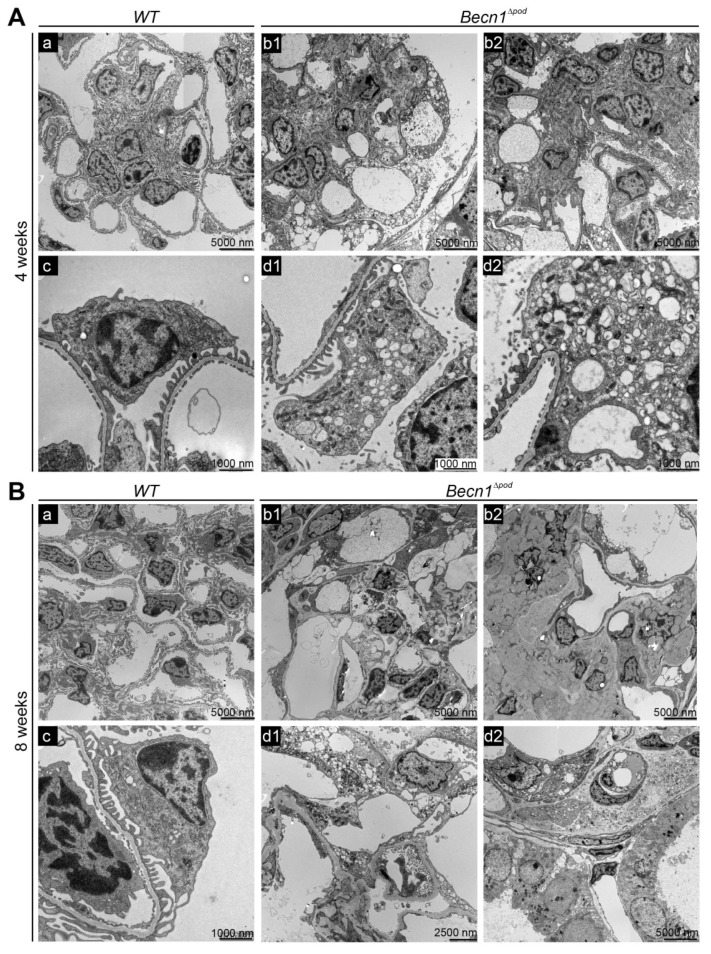
*Becn1*^Δpod^ mice displaying aberrant vesicle accumulation and flattened podocyte foot processes. (**A**) Transmission electron microscopy (TEM) images showing glomeruli (**a**,**b1**,**b2**) and podocytes (**c**,**d1**,**d2**) in kidney sections obtained from 4-week-old *WT* and *Becn1*^∆pod^ mice; (**B**) TEM images showing glomeruli (**a**,**b1**,**b2**) and podocytes (**c**,**d1**,**d2**) in kidney sections obtained from 8-week-old *WT* and *Becn1*^∆pod^ mice.

To further characterize the effect of *Becn1* deficiency on podocytes, extensive ultrastructural studies were performed with transmission electron microscopy (TEM). At the age of four weeks, *Becn1*^∆pod^ mice exhibited accumulated intracellular vesicles in podocytes **(**Figure 4A(b1,b2)). Increased magnification of a single podocyte revealed low vesicle content density and increased vesicle size in the podocyte periphery (Figure 4A(d1,d2)). All the vesicles had a single membrane, indicating that autophagosome accumulation was not a leading cause of the high vesicle number. Despite the initiation of cellular dysfunction, podocyte foot processes remained mostly intact.

At the age of eight weeks, *Becn1*^∆pod^ mice showed mesangial matrix expansion and capillary obliteration in glomeruli (Figure 4B(b1,b2). The podocytes in these mice appeared largely vacuolized, and many showed deterioration, with extensive foot process effacement (Figure 4B(d1,d2)). To further characterize the origin of the accumulating vesicles, and to determine the role played by BECLIN1, primary podocyte cultures of *Becn1*-deficient and the corresponding WT podocytes were established from *Becn1^flox/flox^*;*hNphs2-Cre* mice harbouring a *Tomato/EGFP* allele that resulted in green fluorescence emission from podocytes (Figure S3A). Glomeruli of two-week-old mice were isolated and further cultivated. After four days, a single-cell suspension was obtained from the glomerular outgrowth culture and subjected to FACS, and the pure podocyte population (harbouring the GFP label) was further processed (Figure S3B). Primary podocytes lacking the BECLIN1 mimic in vivo exhibited massive vacuolization (Figure S3C,D). TEM revealed the accumulation of single-membrane vesicles with a low electron density (Figure S3E). These vesicles were located in close proximity to the rough endoplasmic reticulum (RER) (Figure S3E(a2,a3)) and displayed a uniformly round shape (Figure S3, image D). LysoTracker^TM^ staining showed a large lysosomal compartment and many vesicles; however, no uptake of the dye was evident (Figure S3F). Autophagy and autophagic flux were not affected by *Becn1* deletion (Figure S3G).

### 2.4. BECLIN1 Coordinates the TGN and Vesicle Release at the Trans-GOLGI

**Figure 5:**
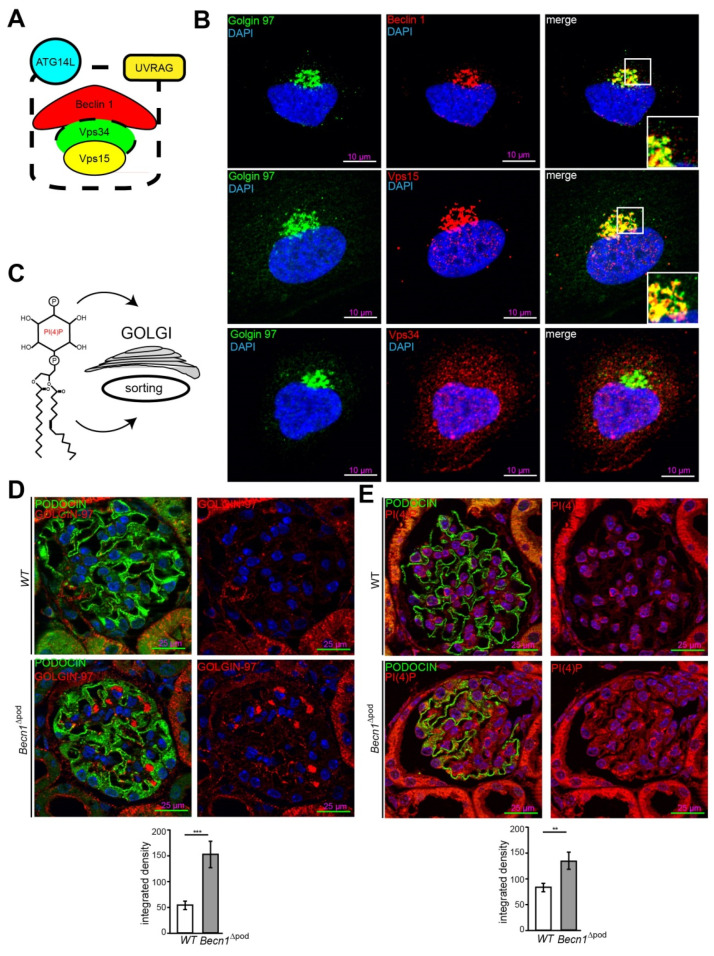
Beclin 1 localizes to the Golgi compartment and regulates Golgi size. (**A**) Schematic showing key interaction partners of the BECLIN1-core complex; (**B**) Immunofluorescence staining for GOLGIN 97 (green), BECLIN1, VPS15 and VPS34 (red) in human podocytes; (**C**) Schematic showing PI(4)P as the key upstream regulator of Golgi size and activity; (**D**) Representative image showing immunofluorescence staining for PODOCIN (green) and PI(4)P (red) in kidney sections obtained from 4-week-old WT and *Becn1*^∆pod^ mice with the integrated density of the GOLGIN 97 signal quantified (n = 8, *** ≤ 0.001); (**E**) Representative image showing immunofluorescence staining for PODOCIN (green) and PI(4)P (red) in kidney sections obtained from 4-week-old WT and *Becn1*^∆pod^ mice the integrated density of the GOLGIN 97 signal quantified (n = 8, ** ≤ 0.001).

**Figure 6:**
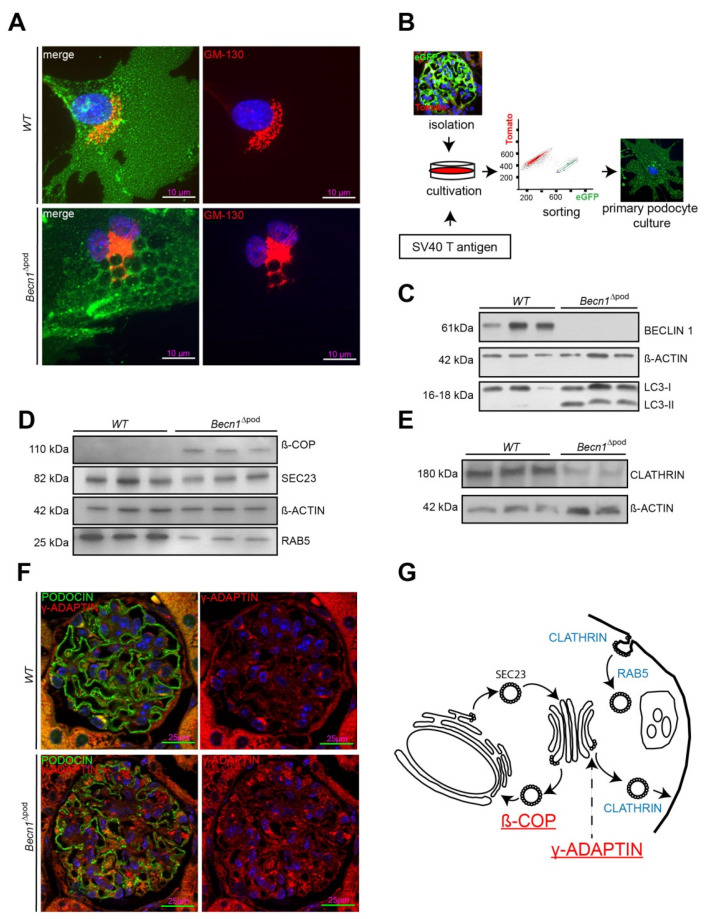
(**A**) Immunofluorescence staining for GM-130 (red) in primary murine *WT* and *Becn1*^∆pod^ podocytes obtained from *Becn1^flox/flox^ x Tomato/EGFP x hNphs2-Cre* and WT *x Tomato/EGFP x hNphs2-Cre* mice (green, EGFP fluorescence after successful *Cre*-mediated recombination); (**B**) Schematic showing the generation of immortalized murine *WT* and *Becn1*^∆pod^ podocytes based on glomerular isolation of *Becn1^flox/flox^*;*Tomato/EGFP*; *hNphs2*-*Cre*+ and WT;*Tomato/EGFP*; *hNphs2*-*Cre*+ with subsequent Adeno-SV40 transduction followed by fluorescence activated cell sorting (FACS); (**C**) Western blot showing the abundance of BECLIN1, ACTIN and LC3 in lysates of *WT* and *Becn1*-deficient immortalized murine podocytes; (**D**) Western blot showing the abundance of ß-COP, SEC23, ACTIN and RAB5 in lysates of *WT* and *Becn1*-deficient immortalized murine podocytes; (**E**) Western blot showing the abundance of CLATHRIN and ACTIN in lysates of *WT* and *Becn1*-deficient immortalized murine podocytes; (**F**) Representative image showing immunofluorescence staining for PODOCIN (green) and γ-ADAPTIN (red) in kidney sections obtained from 4-week-old WT and *Becn1*^∆pod^ mice; (**G**) Schematic showing the abundance of vesicle markers in *Becn1*-deficient cells indicating an accumulation of ß-COP and γ-ADAPTIN (red) and a reduction in CLATHRIN and RAB5 (blue).

To further study the cellular function of BECLIN1 in podocytes, imaging studies of BECLIN1 and assays to identify key interaction partners of the BECLIN1-core complex (the schematic presented in Figure 5A) were performed. Immunofluorescence staining for BECLIN1, VPS34 and VPS15 in human podocytes revealed close colocalization of BECLIN1 and GOLGIN97, suggesting the presence of BECLIN1 in the Golgi network. The same colocalization was observed with BECLIN1 and VPS15. VPS34, however, was also found outside the Golgi compartment (Figure 5B). Given the known impact of VPS15 on phosphoinositide synthesis and catabolism and its distinct localization to Golgi compartment, we assessed levels of phosphatidylinositol 4-phosphate (PI(4)P), the best characterized upstream regulator of Golgi size and activity (shown in the schematic in Figure 5C). Immunofluorescence studies demonstrated increased Golgi size in the podocytes of four-week-old *Becn1*^∆pod^ mice (Figure 5D), and this increase was accompanied by increased levels of PI(4)P (Figure 5E), indicating an enlarged and activated Golgi compartment when BECLIN1 was absent. Accordingly, enucleation of additional accumulating vesicles was observed in close proximity to the GM-130-positive *cis* Golgi in *Becn1*-deficient podocytes, indicating a disruption in Golgi processing and vesicle release from the *trans*-Golgi network (TGN) (Figure 6A). To further study the impact of BECLIN1 on vesicular trafficking, immortalization of primary podocytes was performed by Adeno-SV40 transduction of cells obtained from *Becn1^flox/flox^*;*Tomato/EGFP;hNphs2*-*Cre* and WT;*Tomato/EGFP*;*hNphs2*-*Cre*+ mice and grown in glomerular outgrowth cultures followed by FACS to obtain a pure podocyte fraction (Figure 6B). The ATG-knockout state of the cells and the impact of gene knockout on autophagy were confirmed by assessing the levels of BECLIN1, LC3-I and LC3-II (Figure 6C). *Becn1*-deficient podocytes showed increased levels of ß-COP, a marker of vesicles of retrograde Golgi to ER transport (Figure 6D). In contrast, RAB5, a marker of early endosomes, was decreased, whereas SEC23 levels remained unchanged. CLATHRIN expression was reduced in *Becn1*-deficient podocytes (Figure 6E). Immunofluorescence staining for γ-ADAPTIN, a marker of vesicle formation, clearly revealed γ -ADAPTIN accumulation in the TGN (Figure 6F). In summary, the levels of endocytic pathway markers were reduced in BECLIN1-deficient podocytes, whereas the levels of markers related to anterograde and retrograde Golgi trafficking were massively increased (Figure 6G).

### 2.5. BECLIN1 Is Indispensable for VEGF Secretion and Podocyte-Endothelial Crosstalk

**Figure 7:**
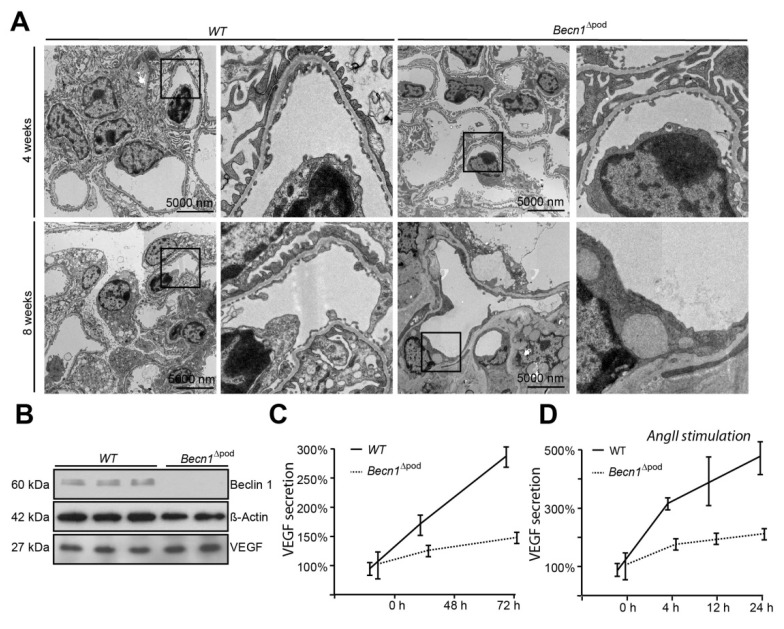

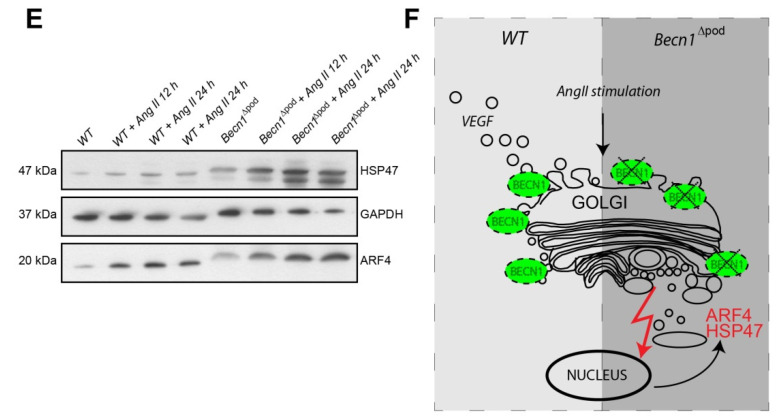
Beclin1 is indispensable for glomerular endothelial integrity and VEGF secretion. (**A**) Transmission electron microscopy (TEM) images of kidney sections obtained from 4-week-old and 8-week-old WT and *Becn1*^flox/flox^ × h*Nphs2*-*Cre* mice; (**B**) Western blot showing the abundance of BECLIN1, VEGF and ACTIN in lysates obtained from immortalized WT and *Becn1*-deficient murine podocytes; (**C**) Relative levels of VEGF in the supernatant of serum-starved WT and *Becn1*-deficient podocytes; (**D**) Relative levels of VEGF in the supernatant of serum-starved WT and *Becn1*-deficient podocytes after angiotensin II (AngII) stimulation (10^−6^ M at the time indicated); (**E**) Western blot showing the abundance of HSP47, ARF4 and GAPDH in lysates obtained from immortalized WT and *Becn1*-deficient murine podocytes with and without AngII stimulation (10^−6^ M); (**F**) Schematic showing the proposed role of BECLIN1 in orchestrating vesicle formation for anterograde Golgi trafficking and VEGF secretion.

Considering the disturbance to intracellular vesicle trafficking observed, we assessed the impact of this disruption on putative podocyte paracrine function. TEM studies of kidney sections of *Becn1*^∆pod^ mouse glomerular endothelium showed loss of fenestration starting when the mice were four weeks old **(**Figure 7A). After eight weeks, fenestration was completely absent, and endothelial cells appeared swollen in the *Becn1*-deficient mice. Immunofluorescence studies confirmed the findings, indicating early endothelial damage in glomeruli by revealing the loss of capillary CD31 positive signals in mice with podocyte-specific BECLIN1 knocked out (Figure S4). To test the hypothesis suggesting that endothelial damage is driven by reduced VEGF secretion by podocytes, basal VEGF production was assessed in immortalized *Becn1*-deficient podocytes and compared to that in WT podocytes, and the analysis revealed no difference in baseline VEGF expression (Figure 7B). VEGF secretion was determined by ELISA performed with cell culture supernatant. *Becn1*-deficient podocytes showed decreased VEGF secretion under basal conditions (Figure 7C) and after stimulation with angiotensin II, an activator of VEGF secretion (Figure 7D). Assessment of the expression of ARF4 and HSP47, important markers of Golgi stress, revealed a massive increase in Golgi stress when VEGF secretion was stimulated in the absence of BECLIN1 (Figure 7E). In conclusion, BECLIN1 emerged as an indispensable factor for Golgi function with subsequent VEGF secretion (Figure 7F).

## 3. Discussion

Autophagy has emerged as a key stress response mechanism in various kidney diseases ranging from acute kidney injury (AKI) to glomerular diseases [4,14,15,16,17,18,19,20]. Accordingly, the expression of a broad spectrum of ATG genes, including *Becn1,* is upregulated in human CKD. In our study, *Becn1*-deficient podocytes showed impaired autophagy and an increase in both SQSTM1 aggregates and LC3- and small GFP-LC3-positive autophagosomes in vivo, indicating successful autophagy initiation despite disruption of the VPS34-BECLIN1 complex, a finding in line with previously reported in vivo findings based on neuronal deletion or deletion of *Becn1* in murine ovaries [21,22]. Our in vitro studies with primary podocytes lacking *Becn1* exhibited only slight autophagic flux impairment, indicating that autophagy can be maintained even when the canonical pathway is disrupted, a finding previously observed with HeLa cells in which transcription-activator-like effector nuclease (TALEN) was used to delete *Becn1* [23]. Accordingly, the phenotype of mice with podocyte-specific deletion of *Becn1* completely differed from that of mice with other podocyte-specific ATG genes knocked out, which led to acquisition of a late-onset phenotype mimicking accelerated ageing [4,24]. In contrast, we report the rapid development of proteinuria and glomerulosclerosis in *Becn1* deficiency, clearly indicating a broad role played by BECLIN1 that encompasses more than autophagy pathway activation. In fact, reports based on yeast studies have suggested that BECLIN1 might play a role in intracellular membrane trafficking [25]; however, evidence in mammals has been lacking. McKnight et al. [3,21] showed that neuronal BECLIN1 mediated early endosome localization and late endosome formation, suggesting a role for BECLIN1 in endocytosis, similar to that observed in *Vps34* deficiency. Gawriluk et al. [22] showed that BECLIN1 is necessary for the clearance of autophagosomes and endosomes and for progesterone synthesis, thereby implicating a key role for autophagy in mammalian reproduction. Studies in Drosophila, however, have suggested another role for BECLIN1 in a secretory pathway. Specifically, flies with a homozygous *Atg6* loss-of-function mutation displayed accumulation of GFP-labelled salivary glue protein four hours after puparium formation, most likely due to secretion defects [26]. Similar findings had been demonstrated in flies deficient in *Vps15*, a close interaction partner of BECLIN1 [27]. However, until now, evidence for a distinct role played by *Becn1* in a secretory pathway in mammals had not been reported.

In our study, we identified BECLIN 1 as a key promoter of podocyte secretory function. Localizing to the Golgi compartment, BECLIN1 critically affects Golgi size and function, thereby governing vesicle formation and membrane trafficking. Electron microscopy of podocytes deficient in *Becn1* clearly revealed accumulation of vesicles, most likely originating from the TGN, as indicated by immunofluorescence experiments. Interestingly, in particular, γ-ADAPTIN—a key component of adaptor complex 1 (AP1)—accumulates in the TGN. The role of AP1 has not been completely resolved; however, previous live-cell imaging studies have shown a clear association of AP1 with transport vesicles originating from the TGN and moving farther outwards to the cell periphery, thereby promoting the transport of membrane proteins from the Golgi to the cell surface [28,29]. Additionally, disruptions to coordinated vesicle release from the TGN has been indirectly highlighted by an increase in retrograde Golgi-to-ER trafficking indicated by the level of ß-COP, an essential component of COPI vesicles that mediate retrograde trafficking [30]. Subsequently, disrupted vesicle formation in cells with *Becn1* deficiency leads to enlarged Golgi and increased Golgi stress (indicated by increased levels for ARF4 and HSP47 expression as described in [31]), upstream mediated by increased levels of PI(4)P, an accumulating substrate precursor of VPS15-VPS34 PI3-kinase complex, a key BECLIN 1-interacting protein [32,33].

To test the functional and clinical impact of our findings, we assessed the effect of *Becn1* deletion on the secretion of VEGF, a candidate protein with a proposed podocyte secretory function. *Becn1* deficiency lowered VEGF secretion, particularly after stimulation with angiotensin II, a key upstream regulator of VEGF secretion [34,35]. Subsequently, endothelial integrity depends substantially on proper BECLIN1 function, as demonstrated by our immunofluorescence and ultrastructural experiments.

Thereby, our data complement previous studies reporting a key role of podocyte derived VEGF in glomerular disease. As mentioned above, VEGF has been shown to be indispensable for glomerular development, whereas, in mature glomeruli, VEGF safeguards endothelial function [2]. Podocyte VEGF secretion is tightly balanced, as reported in previous studies where knock-down, as well as podocyte-specific overexpression of VEGF, caused glomerular damage and proteinuria [36,37,38]. However, in our study the distinct contribution of impaired VEGF secretion on the observed phenotype in podocyte-specific *Becn1* deficiency remains elusive. Our data show that vesicle accumulation and podocyte dysfunction (reflected by albuminuria) precede endothelial damage, whereas later podocyte deterioration and reduced VEGF secretion run in parallel. Future studies might delineate VEGF contribution on the observed phenotype by using a VEGF treatment approach. However, achieving tightly balanced glomerular VEGF levels by external supplementation in the presence of severe proteinuria remains challenging. Moreover, the assessment of urinary VEGF levels in *Becn1*-deficiency is probably not appropriate to clarify VEGF contribution, since VEGF expression in the kidney is not limited to podocytes, and clinical studies report no clear correlation with glomerular pathology [39,40,41,42].

Interestingly, an interplay between autophagy and VEGF has been described in podocytes in which autophagy induction activates VEGF expression, whereas autophagy inhibition decreased VEGF content [43]. In our study, *Becn1-*deficient podocytes still showed ongoing autophagy, and expression levels of VEGF were similar compared to wild type controls in vivo and in vitro, indicating no impact of relevant autophagy-VEGF interplay in our mouse model.

In conclusion, in our study, we describe a novel role for BECLIN1. Considering the in vivo experiments with various transgenic mouse models, the subsequent comprehensive ultrastructural analysis and in vitro experiments with primary podocytes, we identified BECLIN1 as an indispensable component of podocyte secretory and VEGF secretion machinery. Recent studies have identified BECLIN1 protein motifs with autophagy-inducing properties that lead to the development of the tat-BECLIN peptide, which has potential clinical application [44,45]. Our findings open new avenues for developing other BECLIN1-derived therapeutic peptides that promote podocyte-specific VEGF secretion. These peptides may be of high clinical value with distinct applications to mitigate thrombotic microangiopathy (TMA), attenuate renal involvement in preeclampsia and antagonize severe renal side effects after systemic VEGF ablation (as seen in cancer treatment with anti-VEGF antibodies or inhibitors of VEGF receptors) [46,47,48,49].

## 4. Materials and Methods

### 4.1. Mice

*C57BL/6NTac-Becn1tm1a*^(KOMP)Wtsi^/*Cnrm mice* were purchased from the European Mouse Mutant Archive (EMMA). These mice were crossed with FLPo deleter strain mice, and the offspring bearing a *Becn1* flox allele were crossed with *hNphs2-Cre* mice provided by L. Holzman (Renal, Electrolyte and Hypertension Division, University of Pennsylvania School of Medicine, Philadelphia, PA, USA) to generate a podocyte-specific knockout animal. In total, 30 WT and 30 *Becn1*^∆pod^ mice were obtained. The *Becn1*^∆pod^ and WT mice were sacrificed at the time points indicated. To monitor autophagy, the *Becn1^flox/flox^*;*hNphs2-Cre*+ mice were crossed with *Gfp-Lc3* transgenic mice as previously reported [50]. To isolate primary podocytes, *Becn1^flox/flox^*;*hNphs2*-*Cre*+ mice were crossed to *Tomato/EGFP* reporter-expressing mice. *Gt(ROSA)26Sortm4(ACTB-tdTomato, EGFP)Luo/J* mice were purchased from Jackson Laboratory (Bar Harbour, ME, USA). *Becn1^flox/flox^*;*Tomato/EGFP*;h*Nphs2*-*Cre*+ and WT;*Tomato/EGFP*;*Nphs2*-*Cre*+ mice were chosen as the sources for primary cell isolation. All the animal studies were approved by the Committee on Research Animal Care, Freiburg.

### 4.2. Urine and Serum Analysis

Urinary albumin, urinary creatine, serum creatinine and serum urea levels were measured using a fluorimetric albumin test kit (Progen, Heidelberg, Germany, PR2005) and enzymatic colorimetric creatinine and urea kits (Labour + Technik, Wasserburg, Germany, LT-CR0053, LT-UR0010), following the specific manufacturer’s instructions.

### 4.3. Direct Ex Vivo Podocyte Isolation for Knock-Out Confirmation

Direct podocyte ex vivo isolation from 2-week-old *Becn1*^flox/flox^; *Tomato/EGFP;hNphs2-Cre+* and *WT;Tomato/EGFP;hNphs2-Cre+* mice was performed as previously described [51].

### 4.4. Primary Cell Isolation for In Vitro Experiments

Isolation of primary podocytes was performed, as recently described, using sieved glomeruli obtained from 2-week-old *Becn1*^flox/flox^;*Tomato/EGFP*;*hNphs2-Cre+* and *WT;Tomato/EGFP;hNphs2-Cre+* mice, and subsequently FACS was performed to obtain pure podocytes for primary culturing. To generate the primary podocytes, the glomeruli were first isolated from nonproteinuric 12- to 14-day-old pups and cultivated in standard podocyte culture medium (RPMI-1640 from Thermo Fisher Scientific, Waltham, MA, USA, 2187034) supplemented with 10% foetal calf serum (FCS), penicillin/streptomycin (Thermo Fisher Scientific, Waltham, MA, USA, 15140122) and insulin/transferrin/selenite (Roche, Basel, Switzerland, 11074547001). After 5 days of expansion, the cells were trypsinized and transferred into Hanks’ buffered salt solution (Gibco Life Technologies/Invitrogen, Waltham, MA, USA, 14025092) supplemented with 0.1% bovine serum albumin (BSA; Serva, Heidelberg, Germany, 11930). The cell suspension was then sorted by FACS (on a MoFlo^TM^ cell sorter, Beckmann Coulter, Brea, CA, USA), and only EGFP-positive cells were further cultivated. Cultivation was performed using standard podocyte culture medium, and the cells were plated on collagen IV (Sigma-Aldrich, St. Louis, MI, USA, C6745)-coated flasks or dishes (Corning, Corning, NY, USA, 3000 and 353003).

### 4.5. DNA Isolation, PCR and Transcriptome Data

DNA was isolated using a DNeasy kit (Qiagen, Hilden, Germany, 69504) following the manufacturer’s instructions. PCR was performed using a Hot StarTaq Master Mix Kit (Qiagen, Hilden, Germany, 203446) with the following primers (BECN1_frwd, GATGAGGCACTGAGGGCTAC, and BECN2_rev, TAAGAGGGAGAGGGGGCATC). Human transcriptome data were obtained from the Nephroseq database.

### 4.6. Podocyte Staining

Differentiated immortalized human podocytes or primary podocytes obtained from *Becn1* ^flox/flox^;*Tomato/EGFP*;*hNphs2*-*Cre*+ and WT;*Tomato/EGFP*;*hNphs2*-*Cre*+ mice were seeded on collagen-coated cover slips. Twenty-four hours later, the cells were fixed with paraformaldehyde (4% in phosphate-buffered saline (PBS)) and stained with rabbit anti-GOLGIN 97 (Invitrogen, Waltham, MA, USA, PA5-30048), mouse anti-GOLGIN 97 (Invitrogen, Waltham, MA, USA, A-21270), rabbit anti-VPS15 (Novus, St. Louis, MI, USA, NBP1-30463), rabbit anti-VPS34 (Epitomics, Burlingame, CA, USA, 3838-1), rabbit anti-GM-130 (Abcam, Cambridge, UK, ab52649) and rabbit anti-BECLIN 1 (Cell Signaling, Danvers, MA, USA, 3738S) antibodies.

### 4.7. Immunofluorescence Staining

Mouse kidneys were frozen in Tissue-Tek OCT^TM^ (Sakura, Osaka, Japan, SA62550-01) and cyrosectioned at 5 μm (Leica Kryostat). After fixation with 4% paraformaldehyde non-specific protein binding was blocked with phosphate-buffered saline (PBS; Thermo Scientific, Waltham, MA, USA, 10010023) containing 5% BSA. Sections were incubated for 1 h with primary antibodies. After washing with PBS, fluorophore-conjugated secondary antibodies (Invitrogen, Waltham, MA, USA, A-21434, A-11008, A28180) were applied for 30 min. The following antibodies were used to evaluate cryosections: rat anti-NIDOGEN/ENTACTIN (Novus, St. Louis, MI, USA, NBP1-97001), rabbit anti-GFP (Biozol/MBL, Woburn, MA, USA, 598), mouse anti-PI(4)P (Echelon, Bristol, CT, USA, Z-P004), rabbit anti-EEA1 (Cell Signaling, Danvers, MA, USA, 3288) and mouse anti-CD31 (Pharmingen, San Diego, CA, USA, 553370).

For paraffin sections, kidneys were fixed in 4% paraformaldehyde and embedded in paraffin. After cutting in 3 μm sections, the kidney samples were dehydrated, and antigen heat retrieval was performed at a pH of 6. Blocking was performed using PBS containing 5% BSA. Sections were incubated for 1 h with primary antibodies. After washing with PBS, fluorophore-conjugated secondary antibodies (Invitrogen, Waltham, MA, USA, A-21428, A-11008, A28280 or A-21435) were applied for 30 min. The following antibodies were used for paraffin sections: rabbit anti-LC3B (Cell Signaling, Danvers, MA, USA, 2775), guinea pig anti-SQSTM1 (Progen, Heidelberg, Germany, GP62-C), mouse anti-GOLGIN 97 (Invitrogen, Waltham, MA, USA, A-21270), rabbit anti-NPHS2/podocin (Sigma, St. Louis, MI, USA, P0372), guinea pig anti-NPHS1/nephrin (Progen, Heidelberg, Germany, GP-N2) and mouse anti-γ-ADAPTIN (BD, Frankling Lakes, NJ, USA, 610385). Images were taken using a Zeiss fluorescence microscope equipped with a 20× and a 63× water immersion objective lens.

### 4.8. Histology and Electron Microscopy

Kidneys were fixed in 4% paraformaldehyde, embedded in paraffin and further processed for periodic acid–Schiff (PAS) and haemoxylin and eosin (HE) staining. Quantitative assessment of glomerular lesions was performed as previously described by el Nahas [13]. In summary, 2 µm PAS-stained sections from at least 3 animal kidneys were placed on slides and analysed for focal sclerosis (a 1-point score) or total sclerosis (a 2-point score). The sum of the sclerosis scores was divided by the number of glomeruli. This evaluation was performed in blinded fashion and repeated by a second scientist experienced in renal pathology.

For transmission electron microscopy, tissue blocks were washed after fixation in 0.1 M phosphate buffer (PB) and sections (50 µm thick) of the kidney were cut with a vibratome. After incubation in 1% OsO4, the sections were stained with uranyl acetate, dehydrated and flat-embedded in epoxy resin (Durcupan ACM, Fluka, Sigma-Aldrich, Gillingham, UK). Ultrathin sections (40 nm) were cut and analysed with an 80 kV Zeiss Leo transmission electron microscope.

For scanning electron microscopy, glutaraldehyde-fixed kidney samples were dehydrated by sequential incubation in ethanol, followed by incubation in 50:50 ethanol:hexamethyldisilazane. After incubation in 100% hexamethyldisilazane and solvent evaporation, the tissue samples were coated with gold (Zeiss Semco Nanolab7, Polaron Cool Sputter Coater E 5100, Balzer Cpd 020), and imaging was performed with a Leo 1450 VP scanning electron microscope.

### 4.9. Glomerular Isolation, Cell Lysis and Western Blot Procedures

Glomeruli were isolated by perfusion with Dynabeads^TM^ and were subjected to glass-glass homogenization in lysis buffer (RIPA buffer) [52]. Cells were harvested by trypsinization, and lysis was performed using RIPA buffer. The protein concentration was determined with a BCA assay (Pierce Biotechnology, Waltham, MA, USA, 23225). Equal amounts of protein were separated on SDS–PAGE gels. Then, the proteins on the gels were transferred to a polyvinyl difluoride membrane (Trans-Blot^®^ Transfer Pack, Bio-Rad, Hercules, CA, USA, 1704157) with a semidry blotting technique (Trans-Blot^®^ Turbo™ Transfer System, Bio-Rad, Hercules, CA, USA, 1704150). The membranes were blocked in 5% PBS-BSA. The following antibodies were used for Western blotting: rabbit anti-BECLIN 1 (Cell Signaling, Danvers, MA, USA, 3738S), rabbit anti-VEGF (Abcam, Cambridge, UK, 51745), anti-β-actin (Sigma, St. Louis, MI, USA, A5441), rabbit anti-LC3B (Cell Signaling, Danvers, MA, USA, 2775), rabbit anti-β-COP (Abcam, Cambridge, UK, 2899), goat anti-SEC23 (Abcam, Cambridge, UK, 99552), and rabbit anti-RAB5 (Abcam, Cambridge, UK, 218624). Densitometry was performed using LabImage ID software (Kapelan Bio-Imaging).

### 4.10. Cell Culture

Podocytes were kindly provided by Moin Saleem (Bristol, UK). The podocytes were cultured as previously described. Briefly, the podocytes were seeded in RPMI-1640 supplemented with 10% FCS, insulin/transferrin/selenite, penicillin/streptomycin (all from Roche Diagnostics, Mannheim, Germany), pyruvate, 100× minimal essential medium and HEPES buffer (all from Life Technologies, Darmstadt, Germany). When the cells had grown to 60% confluence, differentiation was induced at 37.0 °C for 10–14 days. The cultured human podocytes were fully differentiated before they were used in an experiment.

Primary podocytes obtained from *Becn1*^flox/flox^;*Tomato/EGFP*; *hNphs2*-Cre+ and WT;*Tomato/EGFP*; h*Nphs2*-*Cre*+ mice were cultivated in RPMI-1640 supplemented with 10% FCS, insulin/transferrin/selenite and penicillin/streptomycin.

To expand the number of *Becn1*-deficient primary podocytes, immortalization was induced by transducing glomerular outgrowth cultures of *Becn1*^flox/flox^;*Tomato/EGFP*; *hNphs2*-*Cre*+ and WT; *Tomato/EGFP*;*hNphs2*-*Cre*+ mouse cells with Adeno-SV40. The selection steps were performed using neomycin. After 10 days in culture, the glomerular cells were subjected to FACS to obtain pure podocytes for culturing (GFP-labeled podocytes from *Tomato/EGFP;hNphs2-Cre+*). Gene knockout was confirmed by Western blotting before any experiment was performed. VEGF secretion in the supernatant derived from 10^5^ cells that were serum starved for 4 h was assessed with a Mouse VEGF Quantikine ELISA kit (R&D Systems, Minneapolis, MN, USA, MVV00) following the manufacturer’s instructions (6 replicates per each genotype and timepoint). Angiotensin (Sigma, St. Louis, MI, USA, A9525) was applied at a 10^−6^ M concentration in serum-free media.

### 4.11. Statistical Analysis

The data are expressed as the means ± standard deviation (SD). All experiments were performed at least three times. Statistical comparisons were performed using a two-tailed Student’s t test with the Excel software program. Kaplan–Meier–Meier curves were generated, and log-rank tests were performed using GraphPad Prism software. Differences with a *p* value ≤ 0.05 were considered significant and are marked with *, and differences with a *p* value ≤ 0.01 are marked with **.

## Data Availability

Not applicable.

## Supplementary Materials

Supplementary materials can be found at https://www.mdpi.com/article/10.3390/ijms23073825/s1.
