# Supplementary material for: BECLIN1 Is Essential for Podocyte Secretory Pathways Mediating VEGF Secretion and Podocyte-Endothelial Crosstalk
Source: Int J Mol Sci. 2022 Mar 30;23(7):3825. doi: 10.3390/ijms23073825 (PMC8998849; doi:10.3390/ijms23073825)
Supplement: Supplementary file 1 [file ijms-23-03825-s001.zip › ijms-1631011-Supplementary.pdf]

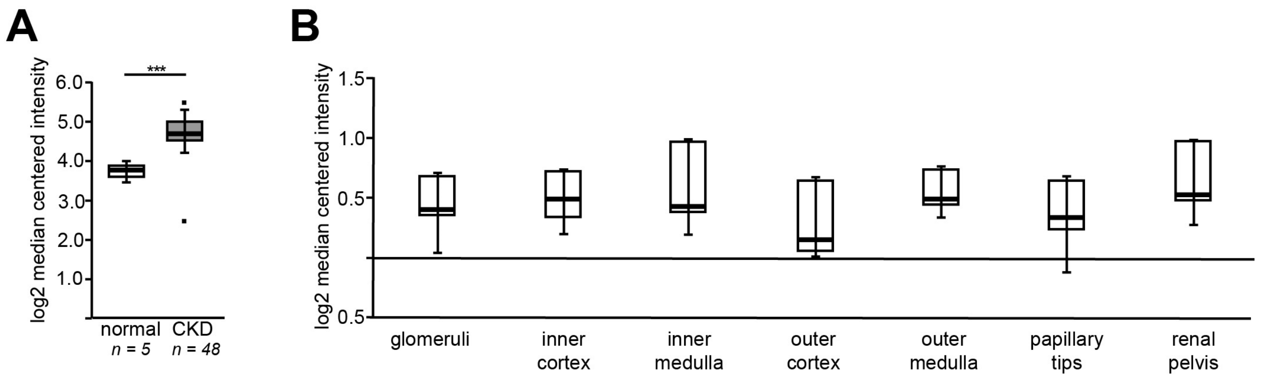

C

Rank	P-value	Fold Change	Gene
172	3.44E-21	2.03	MAP1LC3B
484	1.53E-18	2.12	ZFYVE1
1824	1.50E-13	1.88	ATG5
2066	6.42E-13	1.95	PIK3C3
3121	6.82E-11	1.47	ATG3
3249	1.13E-10	1.86	WIPI1
3498	2.70E-10	1.88	BECN1
4853	8.19E-9	2.13	TMEM49
5285	2.35E-8	1.46	ATG12
7231	2.66E-6	1.46	KIAA0226
7437	4.58E-6	1.37	ATG7
7877	1.21E-5	1.26	ULK1
8177	2.30E-5	1.42	AMBRA1
8232	2.53E-5	1.77	RB1CC1
8634	6.79E-5	1.22	PIK3R4
8822	9.67E-5	1.87	KIAA0831
13056	0.053	1.28	ATG10
13818	0.150	1.10	ATG2A
14096	0.210	1.06	ATG9A
14167	0.233	1.06	MAP1LC3A

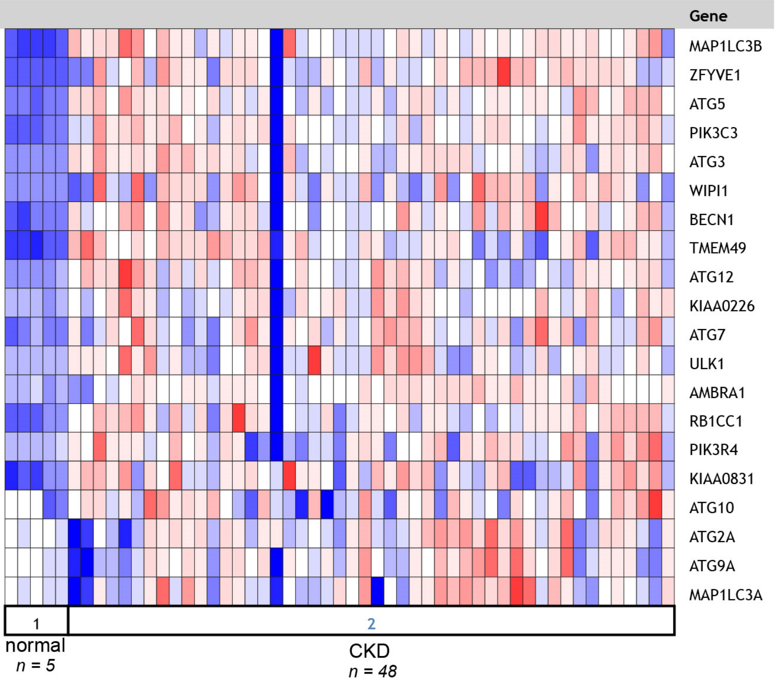

Figure S1. (**A**) Median-centred intensity of BECN1 expression in healthy and diseased kidneys based on Nakawa et al. [12] and obtained from the Nephroseq database; (**B**) Chronic kidney disease (CKD)-associated upregulation of BECN1 expression in different renal compartments based on Nakawa et al. [12] and obtained from the Nephroseq database; (**C**) Expression patterns of ATG genes in healthy and diseased kidneys based on Nakawa et al. [12] and obtained from the Nephroseq database.

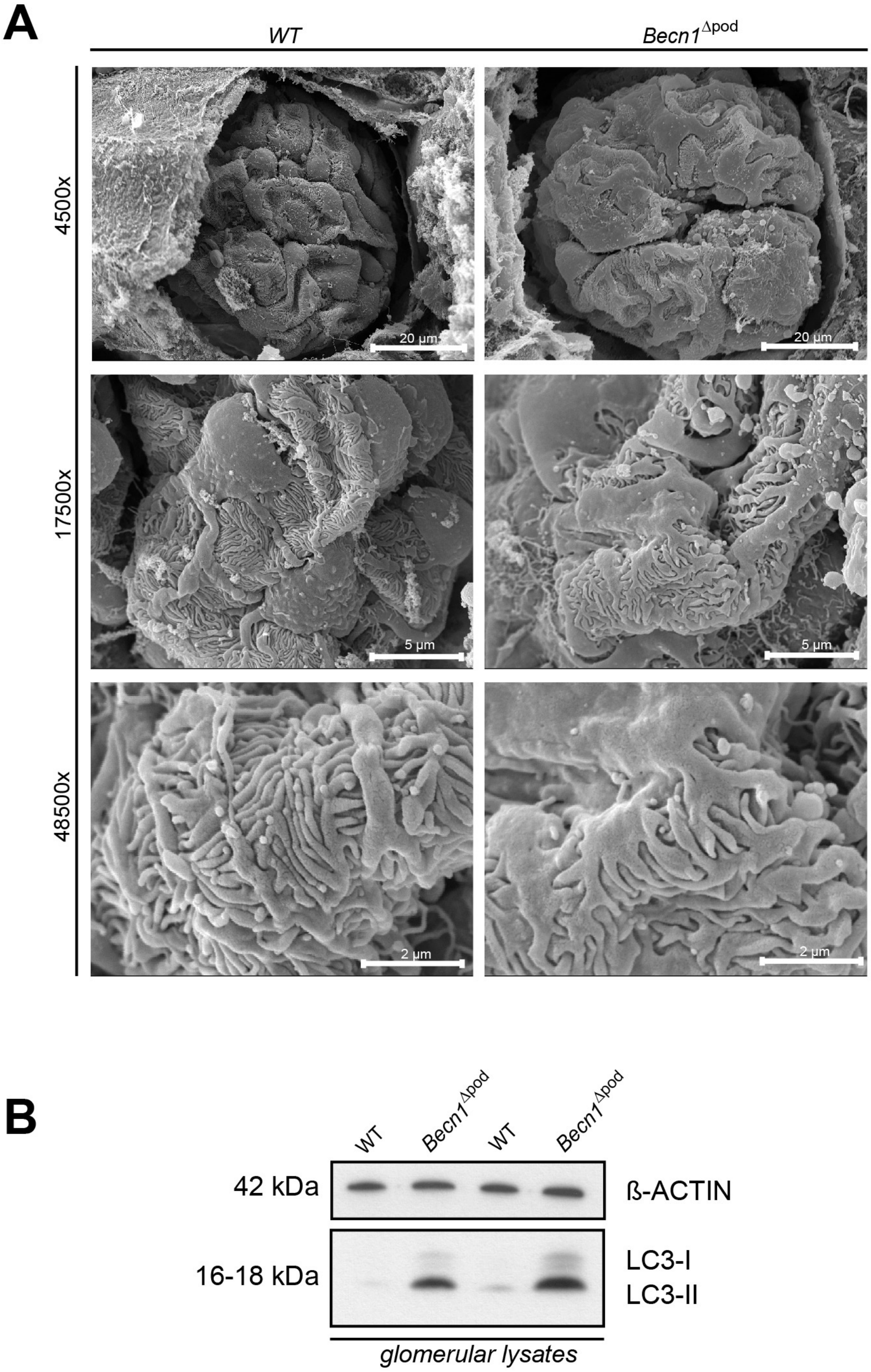

Figure S2. (**A**) Scanning electron microscopy (SEM) images showing kidneys obtained from 4-week-old WT and Becn1flox/flox x hNphs2-Cre mice to visualize foot process formation; (**B**) Western blot showing the abundance of ACTIN and LC3 in lysates obtained from 4-week-old WT and Becn1flox/flox x hNphs2-Cre mice;.

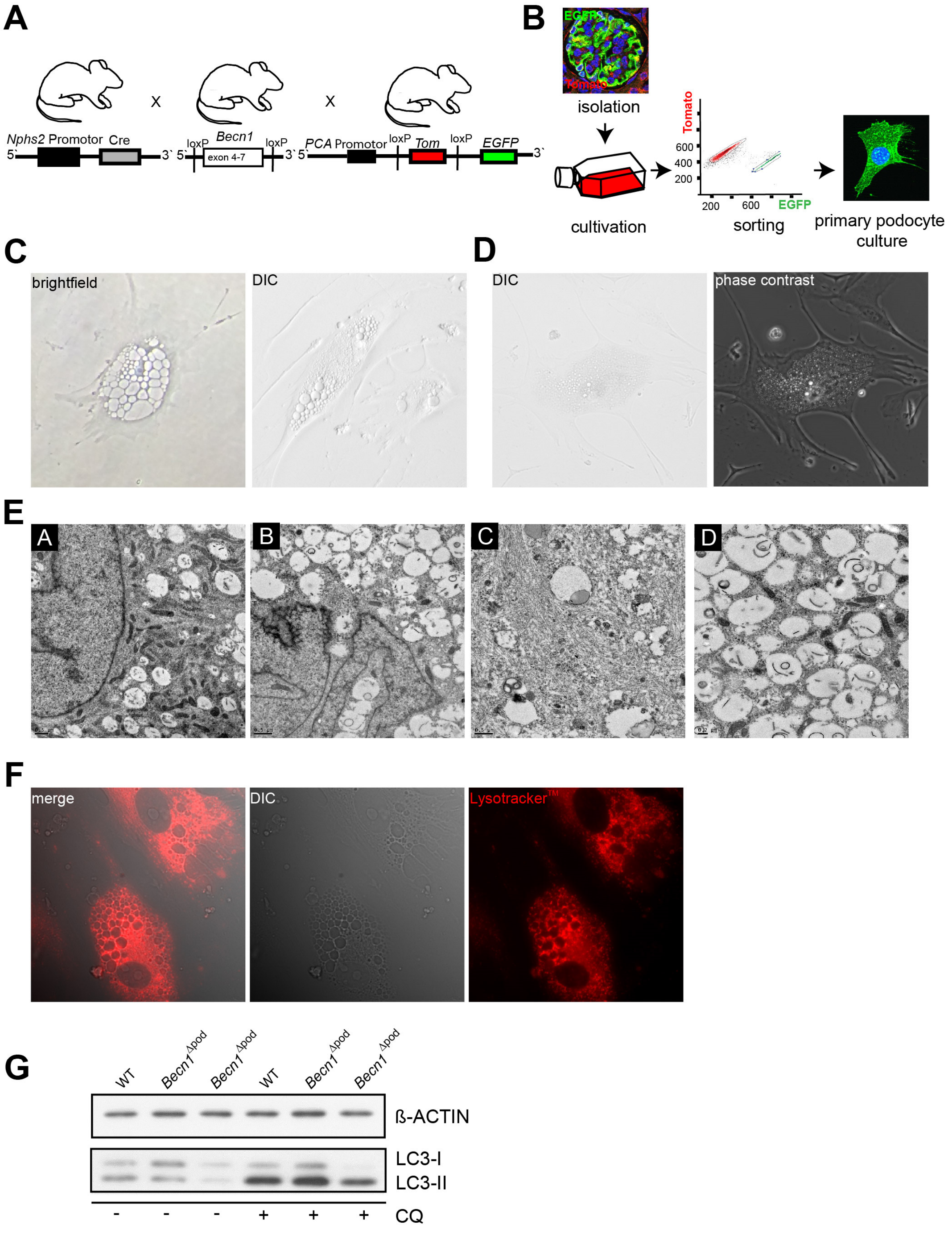

Figure S3. Primary podocytes confirmed in vivo findings showing disrupted vesicle trafficking in Becn1 deficiency. (**A**) Schematic showing Becn1fl/fl mice crossed with hNphs2-Cre mice and Tomato/EGFP-reporter mice to obtain EGFP-labelled WT and Becn1-deficient podocytes; (**B**) Schematic showing primary podocyte culture with glomerular isolation and the outgrowth culture. Fluorescence-activated cell counting was performed to achieve a pure primary podocyte culture; (**C,D**) Primary Becn1-deficient podocytes displaying intracellular vesicle accumulation (brightfield (**C**) and phase contrast (**D**); (**E**) Transmission electron microscopy (TEM) images showing primary Becn1-deficient podocytes intracellular vesicles at different magnifications of intracellular vesicle; (**F**) LysoTrackerTM staining of primary Becn1-deficient podocytes; (**G**) Western blot

showing the abundance of ACTIN and LC3 in primary immortalized WT and Becn1-deficient podocytes with and without chloroquine treatment (100 μM for 2 h);.

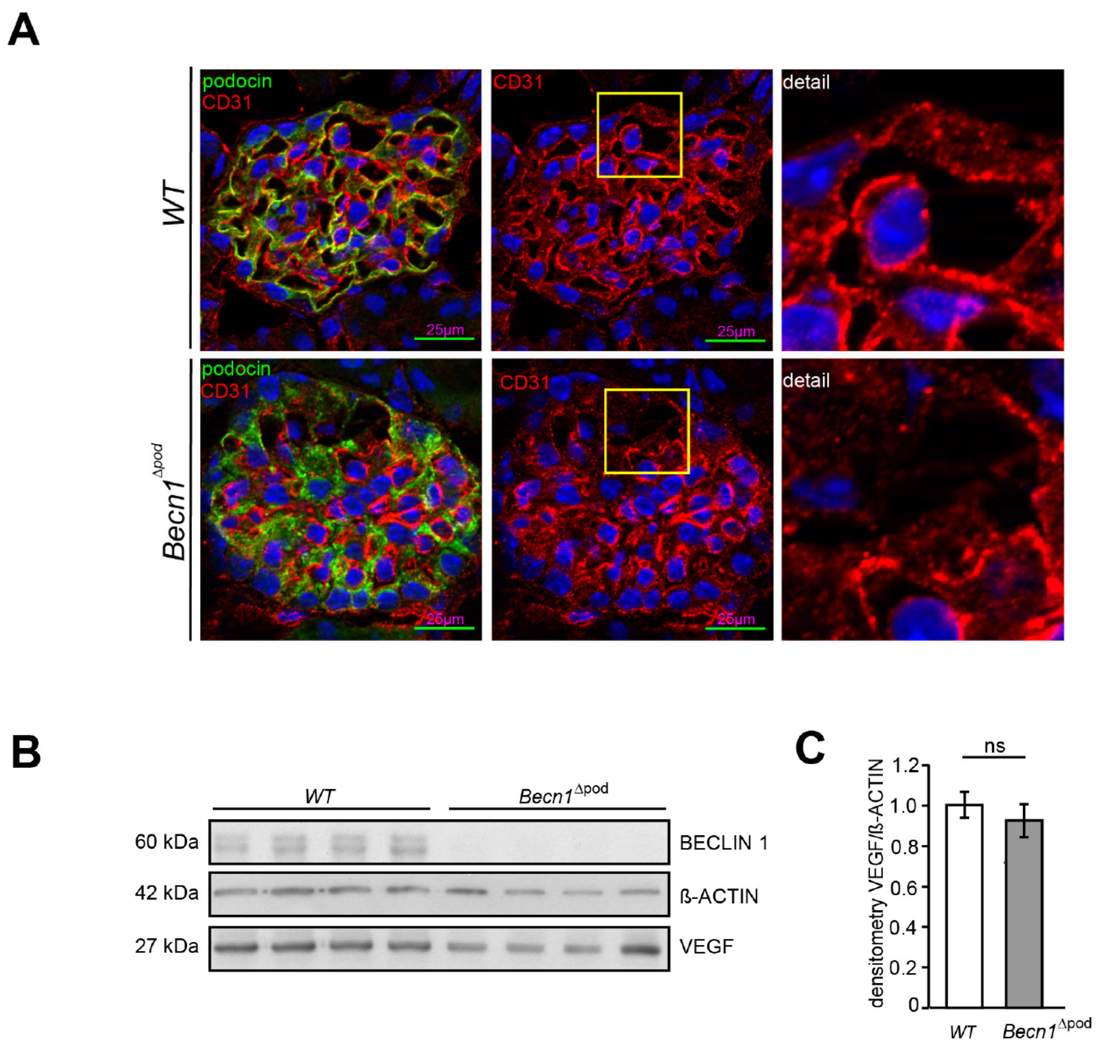

Figure S4. Podocyte-specific deletion of Becn1 leads to endothelial damage in glomeruli. (**A**) Representative image showing immunofluorescence staining for NEPHRIN (green) and CD31 (red) in kidney sections obtained from 4-week-old WT and Becn1Δpod mice; (**B**) Western blot showing the abundance of BECLIN1, VEGF and ACTIN in glomerular lysates obtained from 4-week-old WT and Becn1Δpod mice; (**C**) Densitometry data obtained from samples shown in (**B**) (ns: non-significant).
